# Complete Chloroplast Genome of *Gladiolus gandavensis* (*Gladiolus*) and Genetic Evolutionary Analysis

**DOI:** 10.3390/genes13091599

**Published:** 2022-09-07

**Authors:** Renjuan Qian, Youju Ye, Qingdi Hu, Xiaohua Ma, Jian Zheng

**Affiliations:** Zhejiang Institute of Subtropical Crops, Wenzhou Key Laboratory of Resource Plant Innovation and Utilization, Wenzhou 325005, China

**Keywords:** *Gladiolus gandavensis*, chloroplast genome sequence, comparative analysis, evolutionary genetics

## Abstract

*Gladiolus* is an important ornamental plant that is one of the world’s four most-grown cut flowers. *Gladiolus gandavensis* has only been found in the Cangnan County (Zhejiang Province) of China, which is recorded in the “Botanical”. To explore the origin of *G. gandavensis*, chloroplast genome sequencing was conducted. The results indicated that a total of 151,654 bp of circular DNA was obtained. The chloroplast genome of *G. gandavensis* has a quadripartite structure (contains a large single-copy (LSC) region (81,547 bp), a small single-copy region (SSC) (17,895 bp), and two inverted repeats (IRs) (IRa and IRb, 52,212 bp)), similar to that of other species. In addition, a total of 84 protein-coding genes, 8 rRNA-encoding genes, and 38 tRNA-encoding genes were present in the chloroplast genome. To further study the structural characteristics of the chloroplast genome in *G. gandavensis*, a comparative analysis of eight species of the Iridaceae family was conducted, and the results revealed higher similarity in the IR regions than in the LSC and SSC regions. In addition, 265 simple sequence repeats (SSRs) were detected in this study. The results of the phylogenetic analysis indicated that the chloroplast genome of *G. gandavensis* has high homology with the *Crocus cartwrightianus* and *Crocus sativus* chloroplast genomes. Genetic analysis based on the *rbcl* sequence among 49 *Gladiolus* species showed that samples 42, 49, 50, and 54 had high homology with the three samples from China (64, 65, and 66), which might be caused by chance similarity in genotypes. These results suggest that *G. gandavensis* may have originated from South Africa.

## 1. Introduction

*Gladilous* comprises approximately 265 species, which is one of the largest genera in the family Iridaceae [1]. In addition, *Gladiolus* is a valuable ornamental plant with beautiful colors, and is one of the world’s four most-grown cut flowers [2]. *Gladiolus* is native to Africa and southern Europe. Currently, *G. gandavensis* is found in Cangnan County and Zhejiang Province, China. In Cangnan County, *G. gandavensis* is distributed in Xiaguan town, Mazhan town, and Beiguang Island [3]. *G. gandavensis* likes warm and sunny environments with good ventilation. In its environment, it displays red and yellow flowers and leaves shaped like swords [4]. Therefore, *G. gandavensis* has a high ornamental value, and is mainly used for flower arrangements, bouquets and baskets, as well as in flower beds and as potted plants [5].

The nuclear genome is biparentally inherited, while the chloroplast genome is maternally inherited [6]. In addition, the nuclear genome can be spread by pollen and seeds, and the chloroplast genome can be spread only by seeds in most angiosperm species [7]. Therefore, the chloroplast genome is suitable for identifying plants because of its special characteristics, such as its small size and conservation. Now, the chloroplast genome plays an irreplaceable role in evolution [8], migration [9], and identification [10].

The chloroplast is an important organelle used for photosynthesis and metabolic activities in higher plants and a few algae and prokaryotes [11]. In addition, chloroplasts also play important roles in other aspects of plant physiology and development, which are important for plant responses to light [12,13], heat [14], drought [15,16], salt [17], and other stress [18].

With the popularization and development of NGS technology, chloroplast genome databases are becoming increasingly abundant. Chloroplast genomes of increasing numbers of species have been sequenced, including *Nicotiana tabacum* [19], *Oryza sativa* [20], *Zea mays* [21], *Pinus massoniana* [22], and many other species. In general, there is a typical double-linked loop structure in the chloroplast genome of higher plants, whose sizes range between 120 and 180 kb. The chloroplast genome usually contains a small single-copy (SSC) region, a large single-copy (LSC) region, and an inverted repeat (IR) sequence [23,24].

*G. gandavensis* was only distributed in Cangnan County, China. However, the origin of *G. gandavensis* is unclear. The chloroplast genome was sequenced, and then the cpDNA *rbcl* sequence was used to identify 46 samples of *Gladilous,* to explore the origin of *G. gandavensis*.

## 2. Materials and Methods

### 2.1. Sequencing, Assembly, and Annotation

**Figure 1:**
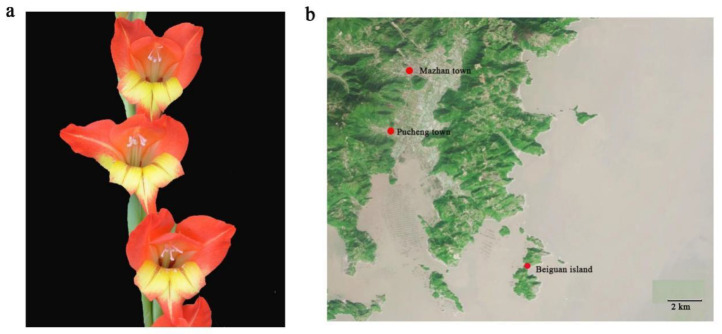
(**a**) The flower with red and yellow colors of *G. gandavensis*. (**b**) The distribution of *G. gandavensis* in Cangnan County (Manzhan town, Pucheng town, and Beiguan Island).

The *G. gandavensis* flowers showed red and yellow pigment, and their distribution was found in Cangnan county, Zhejiang Province, China. In Cangnan county, *G. gandavensis* was distributed in Xiaguan town, Mazhan town, and Beiguang Island (Figure 1). The *G. gandavensis* leaves were collected from Mazhan town (Zhejiang, China, N 27.29°, E 120.43°) for sequencing. An improved extraction method was used to isolate cpDNA from fresh leaves of *G. gandavensis* [25]. A library was constructed with 1 μg of DNA, and a Covaris M220 ultrasonic instrument was used to break the DNA into 300~5500 bp fragments. Afterward, the 3′ ends were polyadenylated and connected to index fragments (TruSeq™ Nano DNA Sample Prep Kit). Library enrichment and PCR amplification for 8 cycles were performed with a 2% agarose gel recycling destination bar (Certified Low Range Ultra Agarose), and then TBS380 (picogreen) was used for quantitation; the materials were mixed according to the data ratio. Then, the generated clusters were subjected to bridge PCR amplification on a CBOT solid phase, and 2 × 150 bp sequencing was performed with an Illumina HiSeq sequencing platform [26].

Original reads were filtered before assembly. SOAPdenovo (version: 2.04, http://soap.genomics.org.cn/soapdenovo.html, accessed on 1 May 2022) was used to assemble the clean data and obtain the optimal assembly results after multiple adjustment parameters [27]. The contigs were obtained by the assembly. The results were partially assembled and optimized according to the reads’ paired ends and overlapping relationships. Then, GapCloser (version: 1.12, http://soap.genomics.org.cn/soapdenovo.html, accessed on 1 May 2022) was used to repair the internal gaps in the sequences, and the redundant sequences were removed to obtain the final assembly sequence [28].

### 2.2. Sequence Analysis

MISA (http://pgrc.ipk-gatersleben.de/misa/misa.html, accessed on 1 May 2022) was used to identify the microsatellite motif [29]. MAFFT v7.310 (https://mafft.cbrc.jp/alignment/software/, accessed on 1 May 2022), which is a multiple sequence alignment software, was used to align the IR sequences between some species in *Gladiolus* [30]. MAUVE was used to locate structural differences among whole-genome alignments [31]. The codon usage bias of (RSCU) was analyzed by (number of codons encoding one amino acid/number of all codons encoding the amino acid)/(1/type of codon encoding the amino acid), namely, the actual frequency of codon usage/frequency of theoretical usage of the codon. Vmatch v2.3.0 (http://www.vmatch.de/, accessed on 1 May 2022) was used to identife the repeat sequence, and the parameter was set to: minimum length (minimum length) = 30 bp, hamming distance (hamming distance) = 3 [32]. KaKs_Calculator v2.0 (https://sourceforge.net/projects/kakscalculator2/, accessed on 1 May 2022) was used to calculate the Ka/Ks, while dnasp5 was used to calculate the pi of every gene [33].

### 2.3. Phylogenetic Analysis with Other Species

For in-depth research, the 8 chloroplast genome sequences of Iridaceae were aligned by MAFFT version 7 [30]. The Iridaceae species and sequences used included *Iris lactea* (MT740331), *Iris sanguinea* (KT626943), *Iris loczyi* (MT254070), *Iris missourensis* (MH251636), *Crous sativus* (MH542233), *Crous cartwrightianus* (MH542231), and *Iris domestica* (MW039136), which was downloaded from https://www.ncbi.nlm.nih.gov/ (accessed on 1 May 2022). Then, the maximum likelihood (ML) method was used to construct the phylogenetic tree [34].

### 2.4. Genetic Evolutionary Analysis

The total DNA of 49 *Gladiolus* (Table 1) samples was extracted using a Plant Genprep DNA Kit (Tiangen, Beijing, China) and quantified using a NanoDrop 2000c instrument (ThermoFisher Scientific, Wilmington, DE, USA) [35,36]. The DNA templates were detected via 1% agarose gel electrophoresis. The ABI-2720 PCR instrument (Applied Biosystems, Waltham, MA, USA) was for PCR amplification. PCR was carried out according to the manufacturer’s protocol. The primers of the chloroplast genome *rbcl* sequence used were ATGTCACCACAAACAGAAAC (forward primer) and TCGCATGTACCTGCAGTAGC (reverse primer). The PCR products were sent to Shanghai Suny Biotechnology Co., Ltd., Shanghai, China, for sequencing. The original sequence data were obtained with Sequencing Analysis 5.2 software. MAFFT was used to align all the sequences. The arithmetic means (UPGMA) method was used to construct the phylogenetic tree [37].

## 3. Results

### 3.1. Characteristics of Chloroplast Genomes

**Figure 2:**
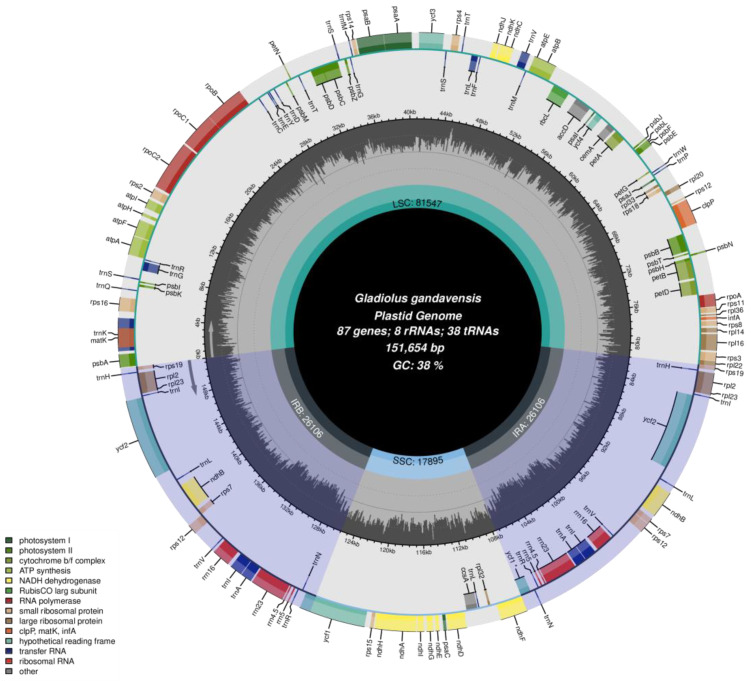
The chloroplast genome of *G. gandavensis*. Genes drawn outside are presented in the clockwise direction, while those inside the circle are presented in the counterclockwise direction. In addition, genes with different functions are represented with different colors.

A circular chloroplast genome with 151,654 bp from *G. gandavensis* was assembled (GenBank accession number: OM304631). The chloroplast genome locations of 1–81,547 bp, 81,548–107,653 bp, 107,654–125,548 bp, and 125,549–151,654 bp were large single-copy (LSC) regions, first inverted repeat (IRa) regions, small single-copy (SSC) regions, and second inverted repeat (IRb) regions, respectively. The contents of the LSCs, IRs, and SSCs in the chloroplast genome were 53.78%, 29.01%, and 17.21%, respectively (Figure 2). In addition, the A, T, C, and G of the nucleotide compositions in the chloroplast genome were 30.79%, 30.88%, 19.2%, and 19.13%, respectively, and the total GC content was 38.34%. Additionally, the chloroplast genome of *G. gandavensis* contains 84 protein-coding genes, 38 transfer-RNA genes (tRNA), and 8 ribosomal RNA genes (rRNA) (Table 2).

### 3.2. Bias of Codon Usage

**Figure 3:**
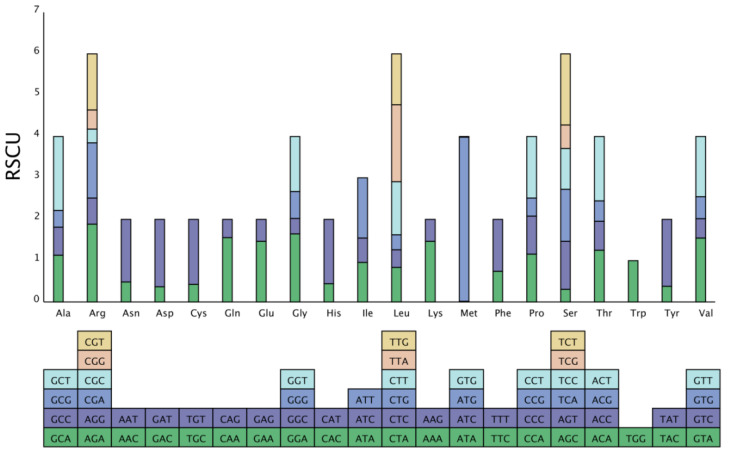
Codon content of 20 amino acids and stop codons in all protein-coding genes of the *G. gandavensis* chloroplast genome. The codons are represented by different colors in the histogram.

Genes from different species or within the same species show different codon usage bias modes, and the current oscillating use of unbalanced codons in biology is called codon usage bias, which helps in better understanding the environmental adaptability and molecular evolution of organisms. In this study, 26,108 codons were identified in all protein-coding sequences. Ile had the highest number (2276) of amino acids, while Met had the lowest number (85) of amino acids. Sixty-eight codons were identified with an RSCU > 1 (Figure 3).

### 3.3. Microsatellite Polymorphisms

**Figure 4:**
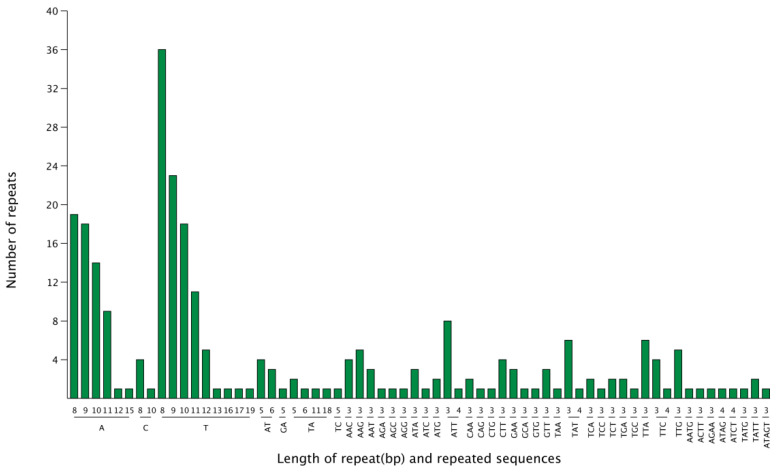
The number of SSRs repeats in the chloroplast genome of *G. gandavensis*. The number of repeats is represented by the green bar charts, and the x-ray represents the length of the repeat (bp) and repeated sequence.

Microsatellite polymorphisms (i.e., simple sequence repeats (SSRs)) were identified in the chloroplast genome of *G. gandavensis,* and distributed in the two different types of regions. There were 171 SSRs located in the LSC regions (64.6%), while 47 (17.7%) and 44 (16.6%) SSRs were located in the SSC regions and IR regions, respectively. In this study, 265 SSRs were identified in the *G. gandavensis* chloroplast genome. Among them, 164 were mononucleotides, 14 were dinucleotides, 78 were trinucleotides, 8 were tetranucleotides, and 1 was a pentanucleotide (Figure 4).

### 3.4. IR Expansion and Contraction

**Figure 5:**
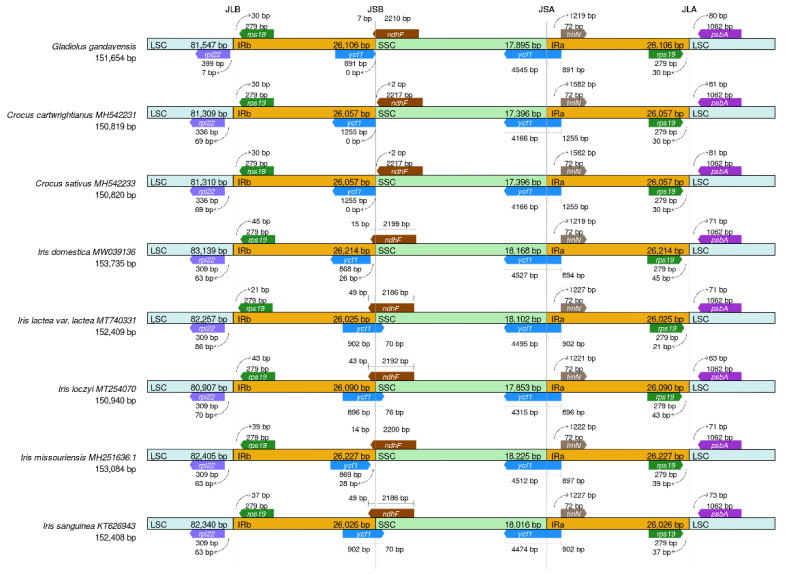
The results of comparing the borders of the LSC, SSC, and IR regions among eight Iridaceae chloroplast genomes: *I. lactea* (MT740331), *I. sanguinea* (KT626943), *I. loczyi* (MT254070), *I. missou-riensis* (MH251636), *C. sativus* (MH542233), *C*. *cartwrightianus* (MH542231), and *I. domestica* (MW039136). Genes located at the IRa/b junctions are represented by colored boxes above (sense), while gene segments represent the boxes below (antisense) the horizontal line.

The IRs serve as integral components of maintaining the stability of the chloroplast genome, as loss of IRs could result in changes in the chloroplast genome [38]. Previous reports have indicated that IR expansion and contraction occur in many plant species [39]. In this study, the IR regions and the junction sites of the LSC and SSC regions in the chloroplast genomes of eight Iridaceae family members (including *G. gandavensis*) were analyzed (Figure 5). The results showed that the IR regions ranged from 150,819 bp in *C. sativus* to 153,735 bp in *I. domestica*. In addition, the *ycf1* gene was located at the SSC/IRa junction in all the chloroplast genomes of different species; however, the *ycf1* gene was missing in the *Iris missouriensis* and *I. domestica,* but was located at the SSC/IRb junction in other chloroplast genomes. Notably, the coding region of *rpl22* was located at the LSC/IRb junction of all the chloroplast genomes, which resulted in the generation of 7, 69, 69, 63, 86, 70, 63, or 63 bp at the LSC/IRb border, respectively.

### 3.5. Phylogenetic Analysis

**Figure 6:**
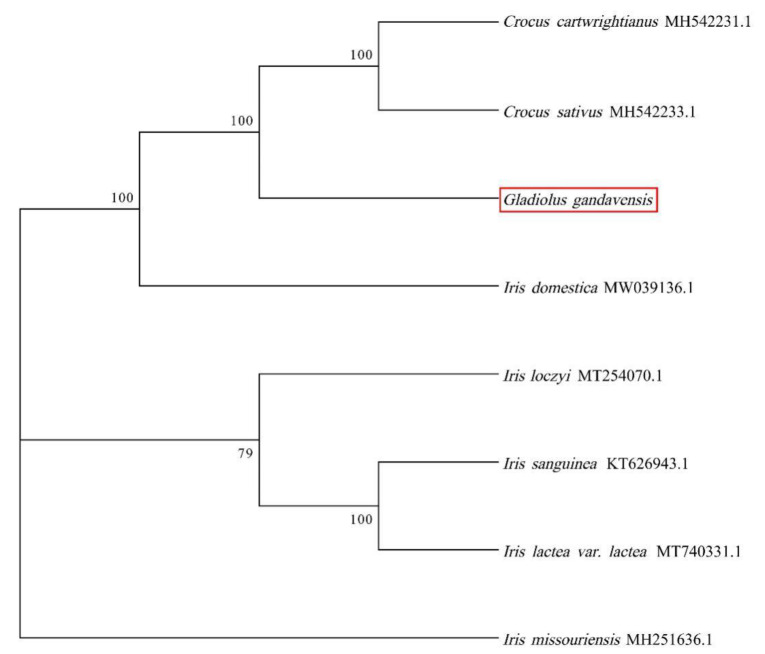
The phylogenetic tree was constructed based on the eight monocotyledons chloroplast genomes: *I. lactea* (MT740331), *I. sanguinea* (KT626943), *I. loczyi* (MT254070), *I. missou-riensis* (MH251636), *C. sativus* (MH542233), *C*. *cartwrightianus* (MH542231), and *I. domestica* (MW039136). The red box represented the chloroplast genome of *G. gandavensis* which was sequenced in this study. The GTRGAMMA model of NJ was used in this study. Bootstrap replicates = 1000.

A phylogenetic tree of eight Iridaceae species was constructed by the GTRGAMMA model. The results showed that *G. gandavensis* had high homology with *C. cartwrightianus* and *C. sativus,* followed by *I. domestica* and other *Iris* species. Therefore, we speculate that *Gladiolus* has high homology with *Crocus* (Figure 6).

### 3.6. Genetic Relationship Analysis of Gladiolus

**Figure 7:**
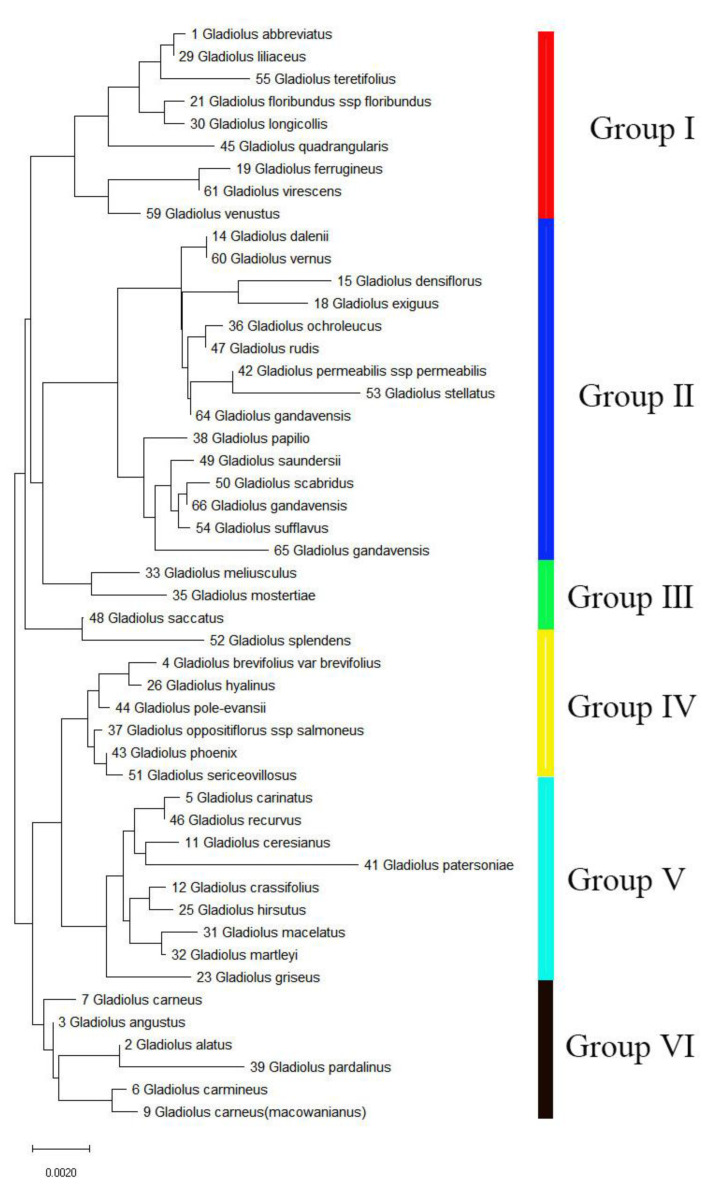
The phylogenetic tree of 49 *Gladiolus* samples was constructed based on the *rbcl* segment. The neighbor-joining (NJ) method was used to constructed the phylogenetic tree. Bootstrap replicates = 1000.

For further study, we amplified and sequenced the *rbcl* segment (cpDNA region) of the 49 *Gladiolus* species to analyze the genetic relationship in *Gladiolus*. Then, we obtained a phylogenetic tree by using the neighbor-joining (NJ) approach (Figure 7). The results showed that the three Chinese species (64, 65, and 66) all clustered into Group II. Specifically, species 66 had high homology with 50 and 54, 65 had high homology with the three species (50, 54, and 65), and 49, and 64 had high homology with 42 and 53. The results indicated that these species may be closely related to the three species.

## 4. Discussion

### 4.1. The Chloroplast Genome of G. gandavensis

The complete chloroplast genome of *G. gandavensis* was assembled. Then, the sequence data were submitted to the NCBI database, under the GenBank number (OM304631). The structure and characteristics of the *G. gandavensis* chloroplast genome were analyzed in this study, and the results were consistent with the traits of most angiosperms. In this study, only the *clpP* and *rps12* genes included two introns. Research on the *clpP* gene indicated that it plays an important role in plant chloroplasts, which are the proteolytic subunits of the ATP-dependent *Clp* protease [40,41,42]. The *rps12* gene is the most unique of all the chloroplast genes and is composed of two parts that are far apart in the genome [43]. Therefore, the study of these two genes would help in understanding the evolutionary process of genes and the genomic characteristics of *G. gandavensis*.

The sequence analysis among *G. gandavensis* and Iridaceae speciesCodon usage bias could avert transcriptional errors in the chloroplast genome by affecting the amino acid functions [44,45,46]. This research showed that the content of bases A and T in mononucleotide SSRs in the chloroplast genome of *G. gandavensis* was 96.95% on average, which was the most frequent. This result is consistent with that of previous reports for most angiosperm chloroplast genomes [47]. Notably, repetitive sequences could be used in phylogenetic studies and genome rearrangements [48]. A comparative analysis of eight Iridaceae plants was conducted. The expansion or contraction of the IR regions in this study indicates that the *ycf1* gene was located at the SSC/IRa and SSC/IRb junctions, and this result will help in exploring the evolution of the chloroplast genome in *G. gandavensis*. Additionally, the phylogenetic tree of eight Iridaceae species indicated that *G. gandavensis* had higher homology with *Crocus* than with *Iris* species. These results show that *G. gandavensis* is closely related to the *Crocus,* which would cause similar molecular evolutionary mechanisms.

### 4.2. The Evolutionary Genetics of G. gandavensis

In addition, we obtained the *rplc* sequence from the 49 *Gladiolus* samples to analyze the evolution of *Gladiolus,* and then, constructed a phylogenetic tree by the NJ approach in this study. The results showed that 42, 49, 50, and 54 samples had high homology with the three Chinese species (64, 65, and 66), which may indicate a close evolutionary relationship. Regain was used with AFLP markers to analyze the genetic relationship of 54 *Gladiolus* cultivars, and the results showed that most of the exotic cultivars as well as indigenous cultivars were closely related to each other. This might be due to a chance similarity in their genotypes [49]. Therefore, we speculate that *G. gandavensis* originated from South Africa. Our results are similar to the previously mentioned studies; however, the specific relationship still needs further exploration. In the future, a study on population genetics, species identification, and conservation biology of *Gladiolus* may be conducted.

## 5. Conclusions

The chloroplast genome of *G. gandavensis* was assembled by Illumina sequencing technology. The sequence information was deposited into the NCBI database under the GenBank number (OM304631). By comparing its structure with that of other Iridaceae species, we found that *Gladiolus* had a higher homology with *Crocus* than with *Iris*. A study on the theoretical relationship among the *Gladiolus* species based on the *rbcl* chloroplast genome sequence will provide reference information for relationship homology, germplasm resource preservation, and sustainable use of these *Gladiolus* species.

## Figures and Tables

**Table 1:** Sample geographic information on *Gladiolus*.

Number	Specific Name	Latin Name	Origin
1	1	*Gladiolus abbreviatus*	South Africa
2	2	*Gladiolus alatus*	South Africa
3	3	*Gladiolus angustus*	South Africa
4	4	*Gladiolus brevifolius var brevifolius*	South Africa
5	5	*Gladiolus carinatus*	South Africa
6	6	*Gladiolus carmineus*	South Africa
7	7	*Gladiolus carneus*	South Africa
8	9	*Gladiolus carneus (macowanianus)*	South Africa
9	11	*Gladiolus ceresianus*	South Africa
10	12	*Gladiolus crassifolius*	South Africa
11	14	*Gladiolus dalenii*	South Africa
12	15	*Gladiolus densiflorus*	South Africa
13	18	*Gladiolus exiguus*	South Africa
14	19	*Gladiolus ferrugineus*	South Africa
15	21	*Gladiolus floribundus ssp floribundus*	South Africa
16	23	*Gladiolus griseus*	South Africa
17	25	*Gladiolus hirsutus*	South Africa
18	26	*Gladiolus hyalinus*	South Africa
19	29	*Gladiolus liliaceus*	South Africa
20	30	*Gladiolus longicollis*	South Africa
21	31	*Gladiolus macelatus*	South Africa
22	32	*Gladiolus martleyi*	South Africa
23	33	*Gladiolus meliusculus*	South Africa
24	35	*Gladiolus mostertiae*	South Africa
25	36	*Gladiolus ochroleucus*	South Africa
26	37	*Gladiolus oppositiflorus ssp salmoneus*	South Africa
27	38	*Gladiolus papilio*	South Africa
28	39	*Gladiolus pardalinus*	South Africa
29	41	*Gladiolus patersoniae*	South Africa
30	42	*Gladiolus permeabilis ssp permeabilis*	South Africa
31	43	*Gladiolus phoenix*	South Africa
32	44	*Gladiolus pole-evansii*	South Africa
33	45	*Gladiolus quadrangularis*	South Africa
34	46	*Gladiolus recurvus*	South Africa
35	47	*Gladiolus rudis*	South Africa
36	48	*Gladiolus saccatus*	South Africa
37	49	*Gladiolus saundersii*	South Africa
38	50	*Gladiolus scabridus*	South Africa
39	51	*Gladiolus sericeovillosus*	South Africa
40	52	*Gladiolus splendens*	South Africa
41	53	*Gladiolus stellatus*	South Africa
42	54	*Gladiolus sufflavus*	South Africa
43	55	*Gladiolus teretifolius*	South Africa
44	59	*Gladiolus venustus*	South Africa
45	60	*Gladiolus vernus*	South Africa
46	61	*Gladiolus virescens*	South Africa
47	64	*G. gandavensis*	China/Beiguan Island
48	65	*G.gandavensis*	China/Mazhan
49	66	*G.gandavensis*	China/Pucheng

**Table 2:** Gene contents of chloroplast genome in *G. gandavensis*.

Category	Gene Group	Gene Contents
Photosynthesis	Subunits of photosystem I	psaA,psaB,psaC,psaI,psaJ
	Subunits of photosystem II	psbA,psbB,psbC,psbD,psbE,psbF, psbH,psbI,psbJ,psbK,psbL,psbM,psbN,psbT,psbZ
	Subunits of cytochrome b/f complex	petA,petB*,petD*,petG,petN
	Subunits of ATP synthase	atpA,atpB,atpE,atpF*,atpH,atpI
	Subunits of NADH-dehydrogenase	ndhA*,ndhB*(2),ndhC,ndhD,ndhE,ndhF,ndhG,ndhH, ndhI,ndhJ,ndhK
	Subunit of rubisco rbcL	rbcL
Self-replication	Small subunit of ribosome	rps11,rps12**(2),rps14,rps15,rps16*,rps18,rps19(2), rps2,rps3,rps4,rps7(2),rps8
	Large subunit of ribosome	rpl14,rpl16*,rpl2*(2),rpl20,rpl22,rpl23(2),rpl32,rpl33, rpl36
	DNA-dependent RNA polymerase	rpoA,rpoB,rpoC1*,rpoC2
	Protease clpP	clpP
	Maturase	matK
	Envelope membrane protein cemA	cemA
	Translation initiation factor infA	infA
	Cytochrome c biogenesis ccsA	ccsA
	Subunit Acetyl-CoA-Carboxylate	accD
	Ribosomal RNAs	rrn16(2), rrn23(2), rrn4.5(2), rrn5(2)
	Transfer RNA	trnA-UGC*(2), trnC-GCA, trnD-GUC, trnE-UUC,trnF-GAA,trnG-GCC*,trnG-UCC, trnH-GUG(2),trnI-CAU(2),trnI-GAU*(2), trnK-UUU*,trnL-CAA(2),trnL-UAA*, trnL-UAG,trnM-CAU,trnN-GUU(2), trnP-UGG,trnQ-UUG,trnR-ACG(2), trnR-UCU,trnS-GCU,trnS-GGA,trnS-UGA, trnT-GGU,trnT-UGU,trnV-GAC(2),trnV-UAC*, trnW-CCA,trnY-GUA,trnfM-CAU
other genes	Maturase	matK
	Protease	clpP**
	Envelope membrane protein	cemA
	Acetyl-CoA carboxylase	accD
	c-type cytochrome synthesis gene	ccsA
	Translation initiation factor	infA
Genes of unknown function	Conserved open reading frames	ycf1, ycf2, ycf2-D2, ycf3, ycf4,

*: one intron; **: two intron.

## Data Availability

Not applicable.
